# Leveraging the predictive power of 3D organoids in dogs to develop new treatments for man and man’s best friend

**DOI:** 10.1186/s12915-023-01799-5

**Published:** 2023-12-29

**Authors:** Karin Allenspach, Yana Zavros, Mohamed Elbadawy, Christopher Zdyrski, Jonathan Paul Mochel

**Affiliations:** 1 Department of Pathology, Precision One Health, College of Veterinary Medicine, University of Georgia, 220 South Riverbend Laboratories, Athens, GA 30530 USA; 2 Department of Cellular and Molecular Medicine, College of Medicine Tucson, University of Arizona, University of Arizona Cancer Center, 1501 N. Campbell Ave, Tucson, AZ 85724 USA

## Abstract

**Recent progress in adult stem cell technology, along with interdisciplinary collaboration in the field of One Health, has accelerated the development of 3D organoid cultures in non-model animals, such as dogs. These advancements have the potential to significantly impact disease modeling and drug development for many diseases shared between man and man’s best friend**.

## 3D organoids and the revolution of cell culture model systems

For decades, the culture of primary epithelial cells from tissues has been constrained by their inherent programming in vivo, resulting in terminal differentiation and eventual programmed cell death within a few days of culture. Traditional cell culture models of epithelial cells typically rely on two-dimensional (2D) cultures of cells of either immortalized or cancer cells. However, these models have inherent limitations in faithfully representing normal or diseased tissues, as they fail to capture the diversity of different epithelial cell types and the three-dimensional (3D) architecture found in living tissues.

In the late 2000s, the discovery of adult stem cells (ASCs) in intestinal epithelial tissues marked a pivotal milestone for the in vitro modeling of human and animal diseases [1]. This ground-breaking discovery paved the way for the development of 3D organoid cultures derived from ASCs which faithfully recapitulate the microarchitecture, physiology, and function of their parent tissue. Within a semi-solid extracellular matrix, ASCs can be cultured, supplemented with tissue- and species-specific growth factors, and self-organize into *mini organs*, known as organoids, which include all the differentiated cell types found in living tissues.

The organoid model has significant advantages over conventional two-dimensional models of epithelial cells, especially for modeling drug efficacy, toxicity, and disease pathophysiology. This is of utmost importance since over 95% of new drugs developed by the biopharmaceutical industry ultimately fail to reach the market [2]. A significant portion of this high attrition rate can be attributed to the use of inaccurate and non-predictive in vitro models, such as 2D cell cultures, which often produce data that does not translate to real-world evidence in humans. Conversely, the 3D organoid model better recapitulates the in vivo biology of tissues and maintains genetic and epigenetic signatures of the original tissues. For instance, the prediction of in vivo toxicity and efficacy of certain drugs can be as high as 80–95% when using 3D organoid models [3].

## The dog as a spontaneous model of human diseases

Rodent models, particularly mice, have been extensively used to study human diseases due to their cost effectiveness, ethical considerations, and easy access to genetically engineered technology. Despite the widespread use of murine models in biomedical research, the translational value of such studies remains limited because they often fail to accurately mimic the underlying human disease. For instance, chronic conditions such as cancer, diabetes mellitus, Alzheimer’s disease, and inflammatory bowel disease are challenging to study in rodents due to their short lifespan, which limits the investigation of environmental contributions to disease development.

Moreover, given the high failure rate of drugs during discovery and development, there is a critical need for more accurate animal models for preclinical studies. This is exemplified by the recent passing of the FDA Modernization Act 2.0, which aims to limit testing of experimental drugs in non-informative animal models, such as rodents before they can be evaluated in clinical trials. Specifically, the FDA Modernization Act allows drug sponsors to use scientifically rigorous, proven non-model animal testing methods or replacement tests when they are suitable. As a result, there is an incentive to use more diversified models, including dogs, which are typically more representative for chronic disease modeling than rodents. This is because dogs have a relatively large body size, longer lifespan, physiology that more closely resembles humans, and a propensity to develop spontaneous, clinical analogues to human diseases.

Cancer has recently gained recognition as one of the diseases that can benefit from using dogs as a natural disease model. Consequently, the population of dogs that naturally develop cancer can serve as a valuable resource for testing new anti-cancer drugs in canine studies before progressing to human clinical trials [4]. This is especially relevant because dog and human cancers share numerous important characteristics, including a similar histological appearance, clinical behavior such as the development of metastatic disease, and genetic and histological heterogeneity within tumors and among patients. Possibly even more important in the context of cancer drug testing is the fact that dogs, much like humans, develop cancers in a complex tumor microenvironment (TME) with an intact immune system. As such, to accurately predict treatment response to immunotherapies, such as immune-checkpoint inhibitors, dogs are considered a superior model compared to most mouse models [5].

## Canine tumor organoids as a precision medicine tool to accelerate drug development for both man and man’s best friend

While the concept of using clinical trials in dogs as a preclinical model for evaluating the safety and efficacy of novel therapeutic drugs holds promise, it does come with some technical challenges. Canine clinical trials, although less expensive than human trials, require similar ethical standards as those used in human research. Additionally, there is a high degree of histological and molecular heterogeneity in canine tumors, similar to humans, and as a result, drug candidates may only benefit a subset of canine patients with specific tumor types. The future of cancer therapy lies in combination treatments, but testing all possible combinations in canine or human clinical trials would be practically impossible. To accelerate the development of patient-specific combination therapies, an ideal solution would be an in vitro model capable of testing multiple drug combinations in a high-throughput fashion with high predictive ability.

3D organoids have shown promise in this regard and outperform 2D models in predicting individual patient responses to drugs. However, the widespread adoption of 3D organoid culture has so far been limited. This is due to multiple factors, for example, because obtaining fresh tissue biopsies, which is necessary for successful organoid development, is not routine in human clinical trial protocols. There are also ethical hurdles when creating bioarchives of human organoid cell lines for research and precision medicine assays, which makes them, to date, a rare resource overall. Canine organoids offer a potential solution as they can accurately model both human and canine diseases. The process of acquiring samples in canine clinical trials is easier, as the protocols are often less rigid than those employed in human clinical trials. In some cases, dogs have a higher prevalence of certain cancers compared to humans, making it possible to create larger bioarchives in a shorter amount of time. This could potentially make canine tumor organoid bioarchives a practical platform for preclinical drug testing, allowing for the identification of promising drug combinations. These combinations can then be further evaluated in both canine and human clinical trials.

## Which canine tumor organoids can be used to model canine and human cancer?

**Fig. 1:**
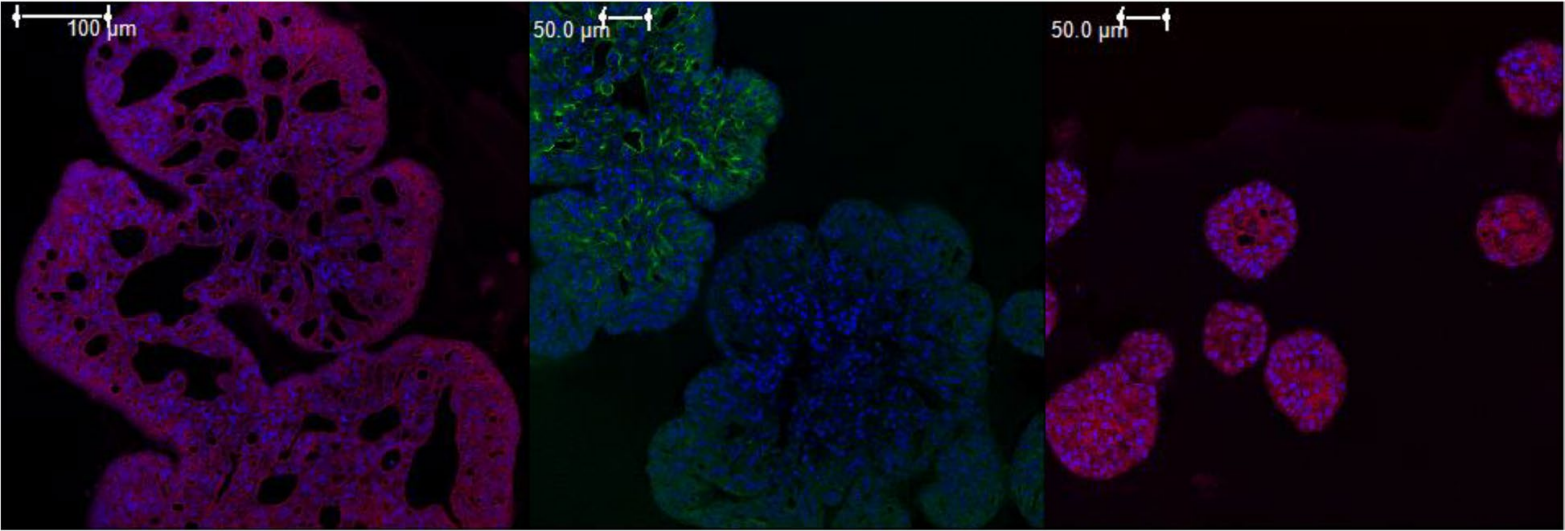
Immunofluorescent staining of canine bladder cancer organoids showing similar expression profiles of dog and human bladder cancer. **A** PAX8 (marker commonly identified in human bladder cancer, in red); DAPI (cell nuclei, blue) overlay. **B** CK5 (cytokeratin marker often positive in human bladder cancer, in red; UPK1 (uroplakin 1 marker, identifies bladder cancer origin, in green; DAPI (cell nuclei, in blue) overlay. **C** P53 (tumor suppressor gene marker often positive in human bladder cancer, in red; DAPI (cell nuclei, in blue) overlay. Scalebars: 100 μm (**A**), 500 μm (**B** and **C**)

**Fig. 2:**
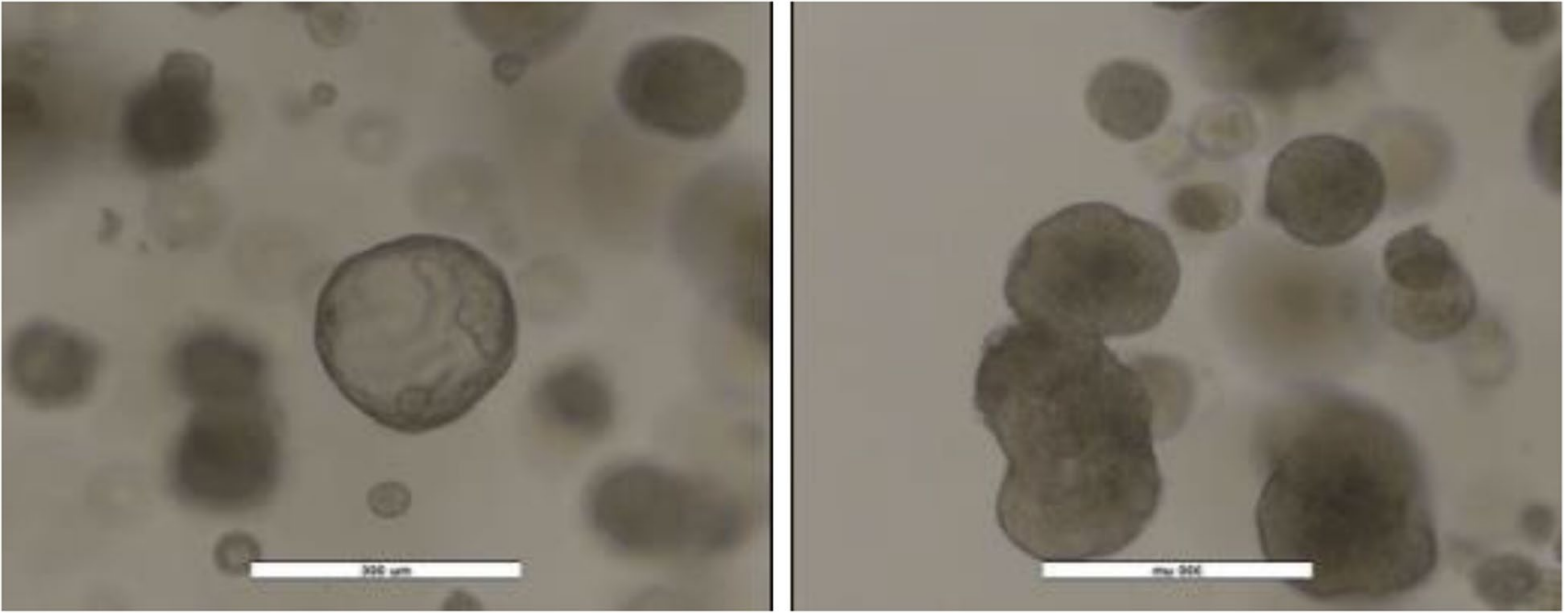
Canine mammary tumor organoids. Tumor organoids derived from a canine malignant mammary tumor on day 3 of culture,  showing spherical structures typical of early organoid cultures (left), and after 3 passages, showing proliferation of the organoids  (right). Scale bar 300 μm

**Fig. 3:**
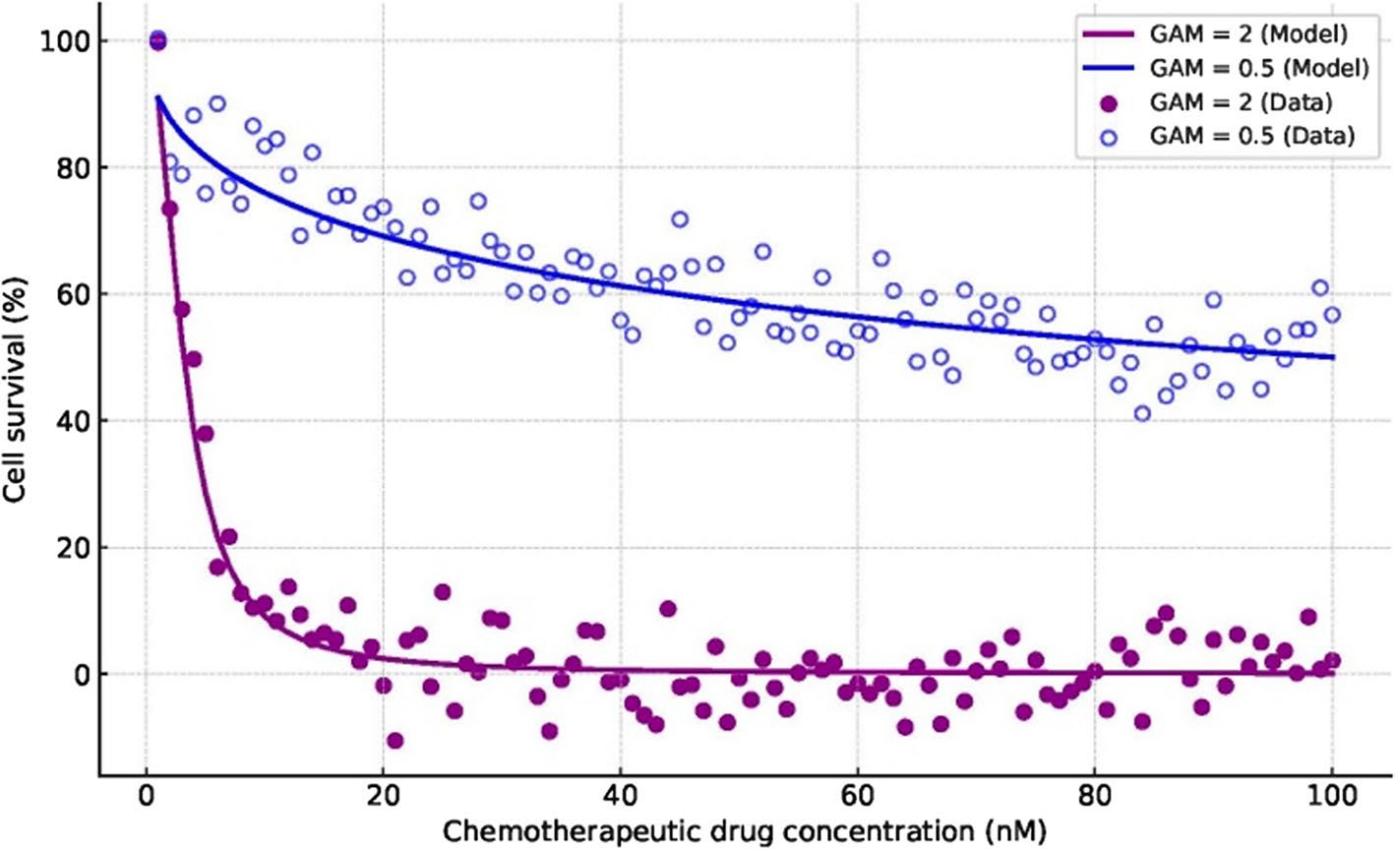
Simulated concentration-response relationship in 3D canine bladder cancer organoids after 24-h exposure to a candidate chemotherapeutic drug. The virtual simulations were based on a Sigmoid Imax model and included two patient sub-populations: (1) an example of a “responder” to the drug with a steep concentration-response curve (closed circles, GAM = 2) and (2) a non-responder who showed minimal changes in cell survival when increasing drug concentrations (open circles, GAM = 0.5). Virtual experimental data are represented as dots, while continuous lines represent model-derived simulations. GAM, gamma parameter (Hill coefficient) from the Sigmoid Imax model

Over the last decade, several canine epithelial cancers have been extensively characterized and shown to be excellent models due to their molecular and histological features as well as their biological behavior. These include tumors such as urothelial carcinoma, mammary carcinoma, bronchial adenocarcinoma, and pituitary tumors. Recently, our lab has made progress in developing robust protocols for culturing organoids derived from several of these canine cancers (see Figs. 1 and 2 for experimental, unpublished data and Fig.  3 for an illustration of concentration–response assays typically performed in the author’s laboratory using a simulated dataset).

In the case of muscle-invasive bladder cancer (MIBC), which is an aggressive form of bladder cancer, dogs are considered to be excellent models of the human disease [6]. Considering that MIBC is relatively rare in humans, the high occurrence rate of these tumors in canines can be leveraged to obtain clinical specimens and conduct canine clinical trials more quickly than would be possible in humans. Canine MIBC organoids have been shown to faithfully recapitulate the histological and molecular characteristics of their parent tumors as well as those found in humans with MIBC [7]. As a result, they could serve as a valuable resource for drug screening for both species.

Mammary carcinomas in dogs have also recently emerged as good models for breast cancer in women. Triple-negative breast cancer (TNBC) is a subtype of mammary carcinoma characterized by the absence of estrogen receptor alpha, progesterone receptor, and human epidermal growth factor receptor 2, which significantly hinders the ability to treat patients with hormone-receptor inhibitor drugs. TNBC is considered one of the most aggressive forms of breast cancer and is associated with poor overall survival. The high intra-tumoral heterogeneity in this subtype of mammary carcinoma also contributes to the lack of effectiveness of traditional treatments such as chemotherapy. Many patients experience drug resistance and high toxicity as a result. Since the majority of dogs diagnosed with malignant mammary carcinoma are neutered, the percentage of dogs presenting with TNBC is higher compared to their human counterparts. This could be leveraged to evaluate new therapeutic drug candidates for TNBC in canine clinical trials in an accelerated timeframe. Additionally, the intra-tumor molecular and histological heterogeneity of these tumors has been found to be similarly high in dogs and humans. As canines’ organoid tumor models accurately reflect the heterogeneity observed in the original tumors [8], this presents an additional opportunity to enable preclinical drug screening of TNBC candidates before conducting formal live testing in dogs and humans.

Non-small cell lung cancer (NSCLC) is the most common type of cancer in people, and it is often diagnosed very late in the disease progression, resulting in an overall poor prognosis. There are many unanswered questions that urgently need to be addressed in the management of NSCLC, such as which subset of patients will respond to chemotherapy, antibody–drug conjugates, tyrosine-kinase inhibitors, immune checkpoint inhibitors, and combinations thereof. Lung carcinoma-derived organoids could once again represent a functional model to help identify answers to these questions. Lung carcinomas in dogs are mostly not induced by smoking, making them an excellent model to study NSCLC in humans, especially in those without a history of smoking, which have been on the rise over the recent decades. Lung cancer organoids derived from dogs have also been described and shown to respond to similar therapeutics as in humans [9].

Lastly, Cushing’s disease (CD) is a serious endocrine disorder caused by an adrenocorticotropic (ACTH)-secreting pituitary neuroendocrine tumor (PitNET). This tumor stimulates the adrenal glands to overproduce cortisol. Despite 40 years of study, the development of therapies directly targeting pituitary tumor cells has only been incremental in decreasing the risk of therapy resistance and tumor recurrence. The rarity of this disease in humans, as well as the absence of a relevant pre-clinical model, has hindered its progress. To this end, dogs develop naturally occurring PitNETs and resulting CD, which closely recapitulate the clinical, histological, immunohistochemical, and treatment response characteristics of the human disease. Notably, CD occurs about 1000 times more frequently in dogs than in humans, making them an excellent model for preclinical evaluation of novel therapeutic drugs. Our lab has recently had success in developing pituitary-derived organoids from dogs, which successfully recapitulated the epithelial architecture of human PitNET organoids and could be used to screen novel drugs before conducting canine clinical trials, selecting only the drugs with the most favorable efficacy and safety profile.

## Future perspectives

The technology for ASC-derived organoid cultures has made significant strides, culminating in several non-model organism organoid culture protocols being published in recent years. Key improvements in this technology include the development of co-culture models, which involve combining epithelial cultures with immune cells, connective tissue, and vasculature. These models will enable in-depth research into specific changes within the tumor microenvironment and the prediction of treatment responses to immunotherapies. Additionally, efforts to standardize techniques and protocols for organoid cultures across different laboratories are essential [10]. Implementing higher throughput systems that allow the simultaneous screening of multiple drugs will greatly enhance the utility of this technology for applications in precision medicine.

## Data Availability

Not applicable.
